# Examination of New Electrocardiographic Repolarization Markers in Diabetic Patients with Noncritical Coronary Artery Disease

**DOI:** 10.1155/2022/5766494

**Published:** 2022-03-12

**Authors:** Müjgan Gürler, Mehmet İnanır

**Affiliations:** ^1^Bolu Abant İzzet Baysal University Faculty of Medicine, Department of Internal Medicine, Bolu, Turkey; ^2^Bolu Abant İzzet Baysal University, Faculty of Medicine, Department of Cardiology, Bolu, Turkey

## Abstract

**Background:**

Diabetes mellitus (DM) is a multisystemic, chronic disease that affects many organs. Coronary artery disease (CAD) is the leading cause of death in patients with DM. The electrocardiogram's new ventricular repolarization parameters can predict mortality and morbidity. The ventricular repolarization indices were examined in diabetic patients with a CAD diagnosis in this study.

**Methods:**

The study group consisted of 84 DM patients (51 males; mean age 58.8 ± 6.6) with noncritical CAD. The control group consisted of 84 DM patients (47 males; mean age 58.7 ± 8.8) with a normal coronary artery. The intervals of QT, QRS, JT, and Tp-e were all measured. Tp-e/QT, Tp-e/QTc, Tp-e/JT, and Tp-e/JTc ratios were determined with QTc, QTd, QTdc, and JTc intervals.

**Results:**

Heart rate (74.4 ± 13.1 vs. 70.0 ± 13.6 bpm, *p*: 0.036), QT (381.0 ± 30.3 vs. 368.6 ± 29.1 ms, *p*: 0.008), QTc (407.5 (359–450) vs. 389 (339–430) ms, *p* < 0.001), QTd (25.1 ± 6.2 vs. 21.9 ± 9.9 ms, *p*: 0.013), QTdc (26.7 ± 6.1 vs. 23.1 ± 10.8 ms, *p*: 0.010), Tp-e (95.7 ± 12.2 vs. 73.6 ± 9.8 ms, *p* < 0.001), JT (293.8 ± 22.0 vs. 283.5 ± 30.9 ms, *p*: 0.014), and JTc (313.6 ± 12.3 vs. 302.4 ± 33.7 ms, *p*=0.005) intervals, and Tp-e/QT (0.25 ± 0.03 vs. 0.20 ± 0.03 ms, *p* < 0.001), Tp-e/QTc [0.23 (0.19–2.33) vs. 0.19 (0.14–0.25) ms, *p*=0.007], Tp-e/JT (0.33 ± 0.04 vs. 0.26 ± 0.04 ms, *p* < 0.001), and Tp-e/JTc (0.30 ± 0.03 vs. 0.24 ± 0.03 ms, *p* < 0.001) ratios were all found to be significantly higher in diabetic patients with noncritical CAD.

**Conclusion:**

In this study, ventricular repolarization markers on the surface ECG were found to be elevated in diabetic CAD patients. These variables may be related to fatal arrhythmic events. To be sure, large-scale, randomized controlled trials are required.

## 1. Introduction 

Coronary artery disease (CAD) is among the major causes of death in the most developed countries [1]. Atherosclerosis of the coronary arteries is the most common cause of CAD. The five major risk factors for cardiovascular disease (CVD) are systolic blood pressure ≥140 mmHg/diastolic blood pressure ≥90 mmHg, total cholesterol ≥240 mg/dL (≥6.222 mmol/L), diabetes mellitus (DM), obesity, and smoking [2, 3].

According to the data of the World Health Organization, there were at least 171 million diabetes patients worldwide in 2000 (2.8 percent of the total world population). Diabetes will affect nearly 330 million individuals by 2030, according to estimates. Diabetes is a condition that affects people all around the world; however, type 2 diabetes is more prevalent in developed countries [4].

DM is a multisystemic, chronic disease that affects many organs. CAD is an important matter in patients with DM. Silent myocardial ischemia is common in DM [5]. Interactions between DM and CVDs are mutual. DM increases the risk of developing CAD by contributing to the development of macrovascular disease and changes in the microcirculation [6]. The presence of DM significantly increases morbidity and mortality in patients with CAD [7]. DM patients mostly die from CVD, including CAD [8]. Therefore, treatment and prevention are essential in patients with DM.

Cardiac autonomic neuropathy (CAN) may develop in DM patients. The underlying pathophysiology is complex and unclear. Parts of its clinical presentation are related to increased sympathetic tone. Conditions such as orthostatic tachycardia, exercise intolerance, and resting tachycardia may be associated with CAN. CAN is related to malignant ventricular arrhythmias and death [9]. Malignant ventricular arrhythmias occur during the repolarization of the heart, and these arrhythmias can be detected by electrocardiography (ECG). A prolonged T-peak to T-end interval on resting ECG has been related to an increased risk of cardiovascular mortality in recent studies [10]. The Tp-e interval is a new ventricular repolarization measure linked to ventricular arrhythmias and sudden cardiac death [11]. The ratio of the Tp-e to the corrected QT interval (QTc) is a reliable indicator of prolonged ventricular repolarization. Although there are studies evaluating ventricular repolarization in DM patients, there is no study evaluating repolarization parameters in diabetic patients with lesions detected on coronary angiography (CAG). The objective of this research was to examine the ventricular repolarization indices in diabetic patients with CAD (diabetic patients with macrovascular complications).

## 2. Material and Methods

### 2.1. Patient Selection

**Figure 1:**
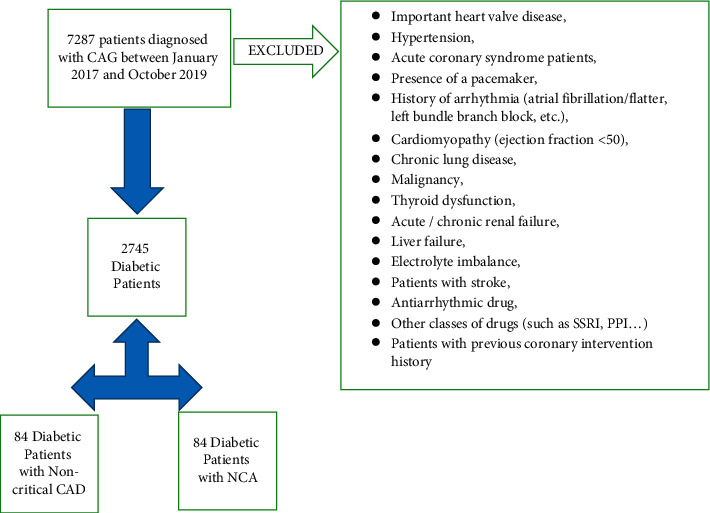
Flow diagram of the study. CAG: coronary angiography, CAD: coronary artery disease, NCA: normal coronary artery.

In the study, 168 patients with DM were included. The DM history of our patients is 1–5 years. The research was designed retrospectively in accordance with the approval of the local ethics committee (2020/86). Medical stories of all participants were obtained from the institutional database. All patients' laboratory and echocardiographic data were recorded. According to the American Diabetes Association's “Medical Care Standards for Diabetes,” all patients were diagnosed with DM [12]. All patients were selected from patients who received CAG. Within the first 15 days before CAG, laboratory parameters, ECG, and echocardiography of all patients were assessed. These measures were used to create exclusion criteria. Flow diagram with exclusion criteria is summarized in Figure 1.

### 2.2. Electrocardiography

12-lead ECG recordings were collected with the Nihon Kohden Cardiofax ECG-1950 VET device at 25 mm/s speed and 10 mm/mV amplitude in the supine position. ECG intervals were measured as shown in the work of Hnatkova et al. [13]. QRS duration, QT interval, JT interval, and Tp-e interval were measured manually with the TorQ 150 mm Digital Caliper LCD device. Tp-e/QT, Tp-e/QTc, Tp-e/JT, and Tp-e/JTc ratios were calculated. Heart rate correction of ECG intervals has been demonstrated in several studies [14, 15]. In our study, QTc, QTdc, and JTc intervals were calculated using Fridericia's formula [15]. The Tp-e interval is not systematically reliant on heart rate, as Andršová et al. [16] pointed out; therefore, heart rate correction should not be applied in clinical Tp-e research. As a result, there was no heart rate correction for the Tp-e interval in our study. Measurements were made by two blinded cardiologists who had no knowledge of the patients. The measurements were measured completely manually. Cohen's Kappa analysis was used to determine intraobserver variability. Cohen's kappa is more suitable for categorical variables [17]. Intraclass variation coefficients were found 0.933 for QRS, 0.969 for QT, 0.976 for JT, and 0.967 for Tp-e. This systematic error among cardiologists was similar for both two groups.

### 2.3. Statistical Analysis

**Figure 2:**
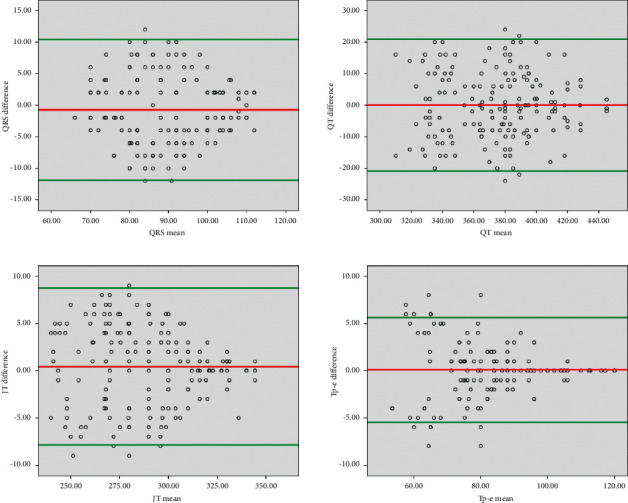
Bland–Altman plots for QRS, QT, JT, and Tp-e.

Statistical analysis was made using an SPSS version 22.0 statistical package (IBM Co., Armonk, NY, USA). Kolmogorov–Smirnov Test and Bland–Altman plots statistics were used to evaluate the normal distribution of data. Quantitative variables were expressed as mean ± standard deviation (SD) and categorical variables as a median (min–max value). Student's *t*-test and chi-square test were used to compare variables. Mann–Whitney's *U* test for variables without normal distribution was used. Bland–Altman plots for each measurement (QRS, QT, JT, and Tp-e) were provided for interpretation (Figure 2). A *p* value of <0.05 was taken into account significantly.

## 3. Results

The patients and the control group were chosen at random for the study. The study group consisted of 84 DM patients (51 males; mean age 58.8 ± 6.6) with noncritical CAD. The control group consisted of 84 DM patients (47 males; mean age 58.7 ± 8.8) with a normal coronary artery. Both groups had similar mean age, sex frequency, hyperlipidemia, BMI, systolic blood pressure (BP), diastolic BP, smoking, left ventricular ejection fraction (LVEF), and hemoglobin A1c (HbA1c) levels (Table 1).

Heart rate (bpm), QT, QTc, QTd, QTdc, Tp-e, JT, and JTc intervals, and Tp-e/QT, Tp-e/QTc, Tp-e/JT, and Tp-e/JTc ratios were all found to be significantly higher in patients with noncritical CAD (Table 2).

## 4. Discussion

The novelty of this study is that patients have known CAD, rather than undetermined CAD. DM is a chronic illness that has a significant effect on the lives and well-being of people, communities, and societies all over the world. The prevalence of DM is progressively rising as a result of aging, rapid urbanization, and an increase in obesity. DM is one of the top ten causes of death in adults, with an estimated four million deaths worldwide in 2017 [19]. By means of oxidative stress, endothelial dysfunction, and vascular remodeling, DM predisposes to serious cardiovascular morbidity and mortality [20]. Studies have shown that DM doubles the risk of vascular outcomes (coronary heart disease, ischemic stroke, and vascular death) regardless of other risk factors [21]. By causing impaired ventricular function and repolarization anomalies, atherosclerosis in patients with DM may cause sudden cardiac death [22, 23]. According to a meta-analysis, DM is associated with a >2-fold increased risk of sudden cardiac death in the general population [24]. CAD is the leading cause of death in patients with DM [20]. According to observational studies and randomized controlled studies, approximately 22 percent of asymptomatic DM patients have silent myocardial ischemia [25]. In around 4% of people with DM, a resting ECG can detect silent myocardial infarction [26].

Atherosclerotic cardiovascular disorders continue to be a major source of all-cause morbidity and mortality around the world, with ventricular arrhythmias being the most prominent predictors of death [27]. In their study, Kahraman et al. [27] showed that ventricular arrhythmic predictors (Tp-e interval, Tp-e/QT, and Tp-e/QTc ratios) increase as one progresses from normal coronary artery to acute coronary syndrome. Also, higher ventricular arrhythmic predictor values were related to more extensive coronary artery disease [28].

DM is one of the most common lifestyle diseases in the world, and it is linked to multisystem damage over time. In the general population, DM is linked to a >2-fold increased risk of sudden cardiac mortality, according to a meta-analysis [29].

In diabetic patients with cardiovascular disease, arrhythmias are one of the leading causes of death. Calculating the Tp-e interval and Tp-e/QTc ratios on an ECG is an easy, practical, and noninvasive approach for predicting mortality. Endocardial, epicardial, and midmyocardial M cells are among the three groups of myocytes present in the myocardium [30, 31]. The electrophysiological properties of these various myocytes demonstrate the variety. Different myocardial electrophysiological properties are affected by the heterogeneity of ventricular repolarization dispersion. Electrical instability in the heart conduction system causes arrhythmias. Ventricular arrhythmias are more likely as a result of this variability [32]. As predictors of ventricular arrhythmia, a variety of parameters have been investigated [33]. These parameters are QT, QTc, QTd, JT, JTc, and Tp-e intervals and their ratios (Tp-e/QT, Tp-e/QTc, Tp-e/JT, and Tp-e/JTc) [10, 34]. The presence of torsadogenic toxicity has long been linked to QTc prolongation [35].

DM is a chronic disease with multiorgan involvement. Major and minor complications are the most important milestones of this disease. One of these important complications is CAD. In diabetic patients, cardiovascular disease is the leading reason for death [36]. Ventricular arrhythmias and sudden death have been linked to DM [37]. The outcome of complications is determined by the course of DM. As a result, an effective DM therapy reduces these risks.

Unlike previous research that looked at repolarization parameters in diabetic patients, we included patients with CAD. Patients with a CAD diagnosis are believed to have a higher risk of ventricular arrhythmic events than patients without a CAD diagnosis. The DM subgroup had higher levels of malignant ventricular arrhythmic predictors, according to our findings.

### 4.1. Limitations

The limited sample size and manual measurement calculations were major disadvantages. Although automated analysis systems have improved the calculation, there are still flaws in the method. Automatic methods may also be preferred for ventricular repolarization parameters.

## 5. Conclusion

In this study, ventricular repolarization markers on the surface ECG were found to be elevated in diabetic CAD patients. These variables may be related to fatal arrhythmic events. To be sure, large-scale, randomized controlled trials are required.

## Figures and Tables

**Table 1:** General characteristics of the study groups.

Baseline characteristics ^*∗*^	Diabetic patients with noncritical CAD (*n* = 84)	Diabetic patients with NCA (*n* = 84)	*P* value
Age (years)	58.8 ± 6.6	58.7 ± 8.8	0.937
Male/female (n)	51/33	47/37	0.534
Systolic BP (mmHg)	122 (100–152)	120 (100–161)	0.254
Diastolic BP (mmHg)	70 (50–94)	80 (60–93)	0.053
LVEF (%)	60 (50–65)	60 (50–65)	0.776
LDL-cholesterol (mg/dL)	112.8 ± 37.7	120.7 ± 35.3	0.160
Triglyceride (mg/dL)	158.5 (73–913)	181 (70–399)	0.625
Total cholesterol (mg/dL)	195.6 ± 40.4	199.2 ± 45.4	0.584
HDL-cholesterol (mg/dL)	47.0 ± 12.9	48.5 ± 12.3	0.427
BMI (kg/m^2^)	32.0 ± 4.7	32.3 ± 5.4	0.340
HbA1c (%)	6.5 (5.2–13.2)	6.7 (5.64–12.8)	0.379

^*∗*^ Quantitative variables were expressed as mean ± standard deviation (SD) and categorical variables as a median (min–max value). BMI: body mass index; BP: blood pressure; CAD: coronary artery disease; HbA1c: hemoglobin A1c; HDL: high-density cholesterol; LDL: low-density lipoprotein; LVEF: left ventricular ejection fraction; NCA: normal coronary artery.

**Table 2:** Electrocardiographic findings of the study population.

Parameters^*∗*^	Diabetic patients with noncritical CAD (*n* = 84)	Diabetic patients with NCA (*n* = 84)	*P* Value
Heart rate (bpm)	74.4 ± 13.1	70.0 ± 13.6	0.036
QRS ms	88.0 ± 12.0	91.4 ± 9.8	0.053
QT ms	381.0 ± 30.3	368.6 ± 29.1	0.008
QTc ms	407.5 (359–450)	389 (339–430)	<0.001
QTd ms	24 (10–40)	22 (6.4–41)	0.013
QTdc ms	26.7 ± 6.1	23.1 ± 10.8	0.010
Tp-e ms	95.7 ± 12.2	73.6 ± 9.8	<0.001
JT ms	293.8 ± 22.0	283.5 ± 30.9	0.014
JTc ms	313.6 ± 12.3	302.4 ± 33.7	0.005
Tp-e/QT	0.25 ± 0.03	0.20 ± 0.03	<0.001
Tp-e/QTc	0.23 (0.19–2.33)	0.19 (0.14–0.25)	0.007
Tp-e/JT	0.33 ± 0.04	0.26 ± 0.04	<0.001
Tp-e/JTc	0.30 ± 0.03	0.24 ± 0.03	<0.001

^*∗*^ Quantitative variables were expressed as mean ± standard deviation (SD) and categorical variables as a median (min–max value). Bpm: beat per minute; ms: millisecond; QT interval: the interval between the start of the QRS complex and the T wave's end; T wave's end: the end of the T wave is determined by the tangent method. The intersection of a tangent to the sharpest slope of the last limb of the T wave and baseline is referred to as the end of the T wave [18]. QTc: corrected QT interval; QTd: QT dispersion (the difference in QT intervals between the maximum and minimum); QTdc: corrected QT dispersion; Tp-e: T-peak to T-end interval; JT: JT interval (the interval between the end of the QRS complex (J point) and the start of the T wave); JTc: corrected JT interval.

## Data Availability

The data used to support the findings of this study are available on request. Medical stories of all participants were obtained from the institutional database. All patients' laboratory and electrocardiographic data were recorded.

## References

[1] Go A. S., Mozaffarian D., Roger V. L. (2013). Heart disease and stroke statistics--2013 update: a report from the American Heart Association. *Circulation*.

[2] Vasan R. S., Sullivan L. M., Wilson P. W. F. (2005). Relative importance of borderline and elevated levels of coronary heart disease risk factors. *Annals of Internal Medicine*.

[3] Wilson P. W. (1994). Established risk factors and coronary artery disease: the Framingham Study. *American Journal of Hypertension*.

[4] Wild S., Roglic G., Green A., Sicree R., King H. (2004). Global prevalence of diabetes. *Diabetes Care*.

[5] Davis T. M. E., Coleman R. L., Holman R. R. (2013). Prognostic significance of silent myocardial infarction in newly diagnosed type 2 diabetes mellitus. *Circulation*.

[6] Sarwar N., Gao P., Kondapally Seshasai S. R. (2010). Diabetes mellitus, fasting blood glucose concentration, and risk of vascular disease: a collaborative meta-analysis of 102 prospective studies. *Lancet (London, England)*.

[7] American Diabetes Association (2018). Cardiovascular disease and risk management: standards of medical Care in diabetes-2018. *Diabetes Care*.

[8] Hammoud T., Tanguay J.-F., Bourassa M. G. (2000). Management of coronary artery disease: therapeutic options in patients with diabetes. *Journal of the American College of Cardiology*.

[9] Vinik A. I., Erbas T. (2013). Diabetic autonomic neuropathy. *Autonomic Nervous System*.

[10] Inanir M., Sincer I., Erdal E., Gunes Y., Cosgun M., Mansiroglu A. K. (2019). Evaluation of electrocardiographic ventricular repolarization parameters in extreme obesity. *Journal of Electrocardiology*.

[11] Panikkath R., Reinier K., Uy-Evanado A. (2011). Prolonged Tpeak-to-tend interval on the resting ECG is associated with increased risk of sudden cardiac death. *Circulation: Arrhythmia and Electrophysiology*.

[12] American Diabetes Association (2019). Care diabetes of medical care in diabetesd 2019. *Diabetes Care*.

[13] Hnatkova K., Toman O., Šišáková M. (2019). Sex and race differences in J-Tend, J-Tpeak, and Tpeak-Tend intervals. *Scientific Reports*.

[14] Hnatkova K., Vicente J., Johannesen L. (2019). Heart rate correction of the J-to-Tpeak interval. *Scientific Reports*.

[15] Luo S., Michler K., Johnston P., Macfarlane P. W. (2004). A comparison of commonly used QT correction formulae: the effect of heart rate on the QTc of normal ECGs. *Journal of Electrocardiology*.

[16] Andršová I., Hnatkova K., Šišáková M. (2020). Heart rate dependency and inter-lead variability of the T peak–T end intervals. *Frontiers in Physiology*.

[17] Ranganathan P., Pramesh C., Aggarwal R. (2017). Common pitfalls in statistical analysis: measures of agreement. *Perspectives in clinical research*.

[18] Malik M., Batchvarov V. N. (2000). Measurement, interpretation and clinical potential of QT dispersion. *Journal of the American College of Cardiology*.

[19] Saeedi P., Petersohn I., Salpea P. (2019). Global and regional diabetes prevalence estimates for 2019 and projections for 2030 and 2045: results from the international diabetes federation diabetes atlas, 9th edition. *Diabetes Research and Clinical Practice*.

[20] Dillmann W. H. (2019). Diabetic cardiomyopathy. *Circulation Research*.

[21] Collaboration ERF (2010). Diabetes mellitus, fasting blood glucose concentration, and risk of vascular disease: a collaborative meta-analysis of 102 prospective studies. *The Lancet*.

[22] Balkau B., Jouven X., Ducimetière P., Eschwège E. (1999). Diabetes as a risk factor for sudden death. *The Lancet*.

[23] Duran M., Ziyrek M., Alsancak Y. (2020). Effects of SGLT2 inhibitors as an add-on therapy to metformin on electrocardiographic indices of ventricular repolarization. *Acta Cardiologica Sinica*.

[24] Campbell P. T., Newton C. C., Patel A. V., Jacobs E. J., Gapstur S. M. (2012). Diabetes and cause-specific mortality in a prospective cohort of one million U.S. Adults. *Diabetes Care*.

[25] Zellweger M. J., Maraun M., Osterhues H. H. (2014). Progression to overt or silent CAD in asymptomatic patients with diabetes mellitus at High coronary risk. *Journal of the American College of Cardiology: Cardiovascular Imaging*.

[26] Valensi P., Lorgis L., Cottin Y. (2011). Prevalence, incidence, predictive factors and prognosis of silent myocardial infarction: a review of the literature. *Archives of cardiovascular diseases*.

[27] Kahraman S., Dogan A., Demirci G. (2021). The association between Tp-e interval, Tp-e/QT, and Tp-e/QTc ratios and coronary artery disease spectrum and syntax score. *International Journal of Cardiovascular Sciences*.

[28] Bektaş O. (2018). The association between coronary artery disease severity and Tp-e Interval, Tp-e/qt ratio parameters including fragmented Qrs. *The American Journal of Cardiology*.

[29] Aune D., Schlesinger S., Norat T., Riboli E. (2018). Diabetes mellitus and the risk of sudden cardiac death: a systematic review and meta-analysis of prospective studies. *Nutrition, Metabolism, and Cardiovascular Diseases*.

[30] Prenner S. B., Shah S. J., Goldberger J. J., Sauer A. J. (2016). Repolarization heterogeneity: beyond the QT interval. *Journal of American Heart Association*.

[31] Gupta P., Patel C., Patel H. (2008). Tp-e/QT ratio as an index of arrhythmogenesis. *Journal of Electrocardiology*.

[32] Xia Y., Liang Y., Kongstad O. (2005). In vivo validation of the coincidence of the peak and end of the T wave with full repolarization of the epicardium and endocardium in swine. *Heart Rhythm*.

[33] Faber T. S., Kautzner J., Zehender M., Camm A. J., Malik M. (2001). Impact of electrocardiogram recording format on QT interval measurement and QT dispersion assessment. *Pacing and Clinical Electrophysiology*.

[34] Inanır M., Gunes Y., Sincer I., Erdal E. (2020). Avaliação de variáveis eletrocardiográficas de despolarização e repolarização ventricular em Diabetes Mellitus Tipo 1. *Arquivos Brasileiros de Cardiologia*.

[35] Hnatkova K., Vicente J., Johannesen L. (2019). Detection of T wave peak for serial comparisons of JTp interval. *Frontiers in Physiology*.

[36] Gregg E. W., Cheng Y. J., Srinivasan M. (2018). Trends in cause-specific mortality among adults with and without diagnosed diabetes in the USA: an epidemiological analysis of linked national survey and vital statistics data. *The Lancet*.

[37] Jouven X., Lemaître R. N., Rea T. D., Sotoodehnia N., Empana J.-P., Siscovick D. S. (2005). Diabetes, glucose level, and risk of sudden cardiac death. *European Heart Journal*.

